# The Biological Behaviors of Rat Dermal Fibroblasts Can Be Inhibited by High Levels of MMP9

**DOI:** 10.1155/2012/494579

**Published:** 2012-04-22

**Authors:** Sheng-Neng Xue, Juan Lei, Chuan Yang, Diao-Zhu Lin, Li Yan

**Affiliations:** ^1^Department of Endocrinology, Sun Yat-Sen Memorial Hospital, Sun Yat-Sen University, 107 Yan Jiang West Road, Guangzhou 510120, China; ^2^Department of Cardiology, Sun Yat-Sen Memorial Hospital, Sun Yat-Sen University, 107 Yan Jiang West Road, Guangzhou 510120, China

## Abstract

*Aims*. To explore the effects of the high expression of MMP9 on biological behaviors of fibroblasts. *Methods*. High glucose and hyperhomocysteine were used to induce MMP9 expression in skin fibroblasts. Cell proliferation was detected by flow cytometry and cell viability by CCK-8. ELISA assay was used to detect collagen (hydroxyproline) secretion. Scratch test was employed to evaluate horizontal migration of cells and transwell method to evaluate vertical migration of cells. *Results*. The mRNA and protein expressions of MMP9 and its protease activity were significantly higher in cells treated with high glucose and hyperhomocysteine than those in control group. At the same time, the S-phase cell ratio, proliferation index, cell viability, collagen (hydroxyproline) secretion, horizontal migration rate, and the number of vertical migration cells decreased in high-glucose and hyperhomocysteine-treated group. Tissue inhibitor of metalloproteinase 1 (TIMP1), which inhibits the activity of MMP9, recovered the above biological behaviors. *Conclusions*. High expression of MMP9 in skin fibroblasts could be induced by cultureing in high glucose and hyperhomocysteine medium, which inhibited cell biological behaviors. Inhibitions could be reversed by TIMP1. The findings suggested that MMP9 deters the healing of diabetic foot ulcers by inhibiting the biological behaviors of fibroblasts.

## 1. Introduction

It is well known that ulcers of diabetic foot are refractory and can cause considerable morbidity and mortality [1]. In recent years, domestic and international studies have found that increased matrix metalloproteinases (MMPs) expression would contribute to the vulnerability of diabetic skin and the refractory nature of diabetic foot ulcers, especially MMP9 [2, 3]. MMPs are proteinases that participate in extracellular matrix macromolecule degradation. MMP9 is one of this growing family. An important mechanism for the regulation of the activity of MMPs is via binding to members of the family of proteins referred to as tissue inhibitor of metalloproteinases (TIMPs). Our previous studies have shown that local abnormal expression of MMP9 in the skin was correlated with skin damage. As compared with nondiabetic rats, the process of wound healing in the skin slowed down in diabetic rat and, at the same time, levels of MMP9 increased significantly, while TIMP1, the tissue-specific inhibitor of MMP9, decreased in the diabetic group [4–6].

It is widely recognized that high levels of MMP9 slow down the healing of diabetic foot ulcers by excessive degradation of extracellular matrix, growth factors, growth factor receptors, integrins, and their receptors, as well as increasing the local inflammatory response in the wound [7–9]. It is still unknown whether MMP9 can influence the biological behaviors of skin fibroblasts and affect wound healing. Skin fibroblasts play important roles in wound repairing. Anything that can affect their biological properties will ultimately affect wound healing. To investigate the mechanisms of MMP9 in diabetic foot wound healing, rat skin fibroblasts were cultured in high glucose and hyperhomocysteine medium [10–12]. Changes of biological behaviors of skin fibroblasts were observed before and after using the exogenous TIMP1.

## 2. Methods

### 2.1. Cell Culture and Grouping

Fibroblasts (CRL-1213, ATCC, USA) were cultured in DMEM (Gibco, USA) containing 10% fetal bovine serum (FBS, Hyclone, USA) in a CO_2_ incubator (37°C, 5% CO_2_). After 24-hour serum deprivation (0.5% FBS), fibroblasts were cultured in DMEM containing 10% FBS at three different conditions (according to the grouping) for 6 hours in a CO_2_ incubator (37°C, 5% CO_2_).

Grouping: (1) Control group was cultured in DMEM containing normal glucose (5.5 mmol/L). (2) Model group was cultured in DMEM containing glucose (22.0 mmol/L) and homocysteine (100 *μ*mol/L) [10–12]. (3) TIMP1 group was cultured in DMEM containing glucose (22.0 mmol/L), homocysteine (100 *μ*mol/L), and TIMP1 (100 *μ*g/L, R and D Systems, Minneapolis, USA).

### 2.2. Determination of MMP9 Levels

MMP9 mRNA expression was determined by real-time polymerase chain reaction (real-time-PCR) [13]. Total RNA was extracted by Trizol (Invitrogen, Carlsbad, CA, USA) from cells. To quantify MMP9 mRNA levels by real-time-PCR, GAPDH was used as the internal control. Primers were designed by Invitrogen Corporation (Carlsbad, CA, USA) according to the sequences of rat MMP9 and GAPDH in GeneBank. Rat MMP9 primers were sense, 5′-TCCAGTAGACAATCCTTGCAATGTG-3′; anti-sense, 5′-CTCCGTGATTCGAGAACTTCCAATA-3′. Rat GAPDH primers were sense, 5′-GGCACAGTCAAGGCTGAGAATG-3′; anti-sense, 5′-ATGGTGGTGAAGACGCCAGTA-3′. Amplification was performed according to the instructions of the SYBR PrimeScript PCR Kit (Takara, Kyoto, Japan). Briefly, SYBR Premix Ex TaqTM (2×) 10 *μ*L, PCR Forward Primer (10 *μ*M) 0.4 *μ*L, PCR Reverse Primer (10 *μ*M) 0.4 *μ*L, ROX Reference Dye (50×) 0.4 *μ*L, and cDNA template 2.0 *μ*L were added into a microfuge tube along with distilled water to make a total volume of 20.0 *μ*L. The PCR reactions were performed in a LightCycler 480 real-time-PCR system (Roche, Basel, Switzerland), with denaturation at 95°C for 30 sec, then 40 cycles of 95°C for 5 sec followed by elongation at 60°C for 20 sec. In order to ensure equal sample sizes, the real-time-PCR for GADPH was performed at the same time. Melting curve analysis was conducted to ensure the specificity of amplification, the products were quantitated by the 2^−ΔΔCT^ method, and the difference between the Ct values of MMP9 and the corresponding value of GADPH in each sample was used as the relative MMP9 Ct value.

MMP9 protein was measured by ELISA assay. At the end of cells culture, supernatants were collected. Double-antibody sandwich ABC-ELISA was used to detect protein expression of MMP9. Operation was performed according to the instructions of the rat MMP9 ELISA kit (Uscn Life Science Inc., Wuhan, China).

MMP9 protease activity in cell culture supernatants were assessed by gelatin zymography [14]. Equal aliquots of conditioned culture media from an equal number of cells were fractionated using precast zymogram gels containing gelatin, according to the manufacturer protocol (GuangDong Chemicalreagent Inc., Guangzhou, China). After electrophoresis, gels were incubated in renaturing buffer (50 mmol/L Tris-HCl (pH 7.4), 2% (vol/vol) Triton X-100) for 30 min at room temperature and then incubated in developing buffer (50 mmol/L Tris-HCl (pH 8.0), 2.5 mmol/L CaCl_2_, 0.02% (wt/vol) Brij-35) for 72 h at 37°C. Lytic bands corresponding to the latent form of MMP9 (92 kDa) were analyzed as total activity and visualized by staining with 0.5% (wt/vol) Coomassie Brilliant Blue solution. The gels were photographed, followed by analysis using Image Quant 5.2 software.

### 2.3. Proliferation of Cells

Proliferation of cells was assessed by flow cytometry [15]. Cells were collected into the dedicated tube for flow cytometry and centrifuged for 6 min (2500 r/min), and supernatant was discarded. Cells were resuspended with 1 mL PBS. Then 300 *μ*L of cell suspension was added into 700 *μ*L ice-cold ethanol drop by drop, fixed overnight at 4°C in the dark. On the next day, suspension was centrifuged for 10 min (2500 r/min) and supernatant was discarded. Cells were resuspended again with 500 *μ*L PBS containing RNase A (100 U/mL), incubated for 30 min at 37°C. Then ethidium bromide (2 mg/mL) was added to a final concentration of 50 *μ*g/mL and incubated for 30 min in the dark. Cells cycle was detected by standard procedures of flow cytometry, and the S-phase cell ratio and proliferation index were calculated at the same time. S-phase cell ratio is S/(G0/G1 + S + G2/M); proliferation index is (S + G2/M)/(G0/G1 + S + G2/M).

### 2.4. Viability of Cells

Viability of cells was assessed by CCK-8 (Dojindo Laboratories, Kumamoto, Japan) [16]. Cells were inoculated to 96-well plates according to the density of 2.0-3.0 × 10^3^ cells for each well. Premixed CCK-8 and medium (10 *μ*L : 100 *μ*L) were added into 96-well plates, and cells were then incubated for 0.5–1 h at 37°C. The values of A450 were obtained with the 3,550 automatic detector from Beckman (Brea, CA).

### 2.5. Collagen (Hydroxyproline) Secretion

Collagen (hydroxyproline) secretion was detected by ELISA. Hydroxyproline was measured according to the instructions of the rat hydroxyproline ELISA kit (Xinqidi Biological Technology Co., Wuhan, China). The concentration of hydroxyproline was calculated according to the A450 value obtained with the 3,550 automatic detector from Beckman (Brea, CA).

### 2.6. Horizontal Migration of Cells

Horizontal migration was assessed by scratch test [17]. Cells cultured in 6-well plates were scratched with a small tip along the ruler. Washing the scratch area was repeated with PBS until the cells in this area were removed thoroughly. Adding the purpose medium and culturing 6 h, we chose five different horizons under the inverted microscope and measured the distance between cells at 0 h and 6 h after scratching, taking the average for horizontal migration rate. Horizontal migration rate is (width at 0 h − width at 6 h)/width at 0 h × 100%.

### 2.7. Vertical Migration of Cells

Transwell was used to evaluate the vertical migration of cells [18]. Cells in the logarithmic growth phase were suspended by purpose medium containing 0.5% FBS after conventional digestion. 100 *μ*L cell suspensions (1.0 × 10^5^/mL) were added into the upper chamber, and 500 *μ*L purpose medium (according to the grouping) containing 10% FBS was added into the lower chamber. After 16 h of culture, the upper chamber was removed, fixed for 30 min with 4% paraformaldehyde, and stained for 15 min with crystalline violet. We selected four visions randomly to count the number of cells moved to the lower of the membrane under inverted microscope, taking the average for the number of vertical migration cells.

### 2.8. Statistical Analysis

Data are presented as mean ± SEM. Comparisons were made by one-way ANOVA followed by Student-Newman-Keuls post hoc analysis. Data were analyzed with Microsoft Excel 2003 (Microsoft Inc., Seattle, WA, USA) and SPSS 12.0 for Windows (SPSS Inc., Chicago, IL, USA). Statistical analyses were performed using the average results of three repeated experiments under identical conditions. A *P* value <0.05 was considered statistically significant. Differences were considered significant if *P* < 0.05.

## 3. Results

### 3.1. Establishment of the Fibroblast Cell Model of High MMP9 Expression

**Figure 1:**
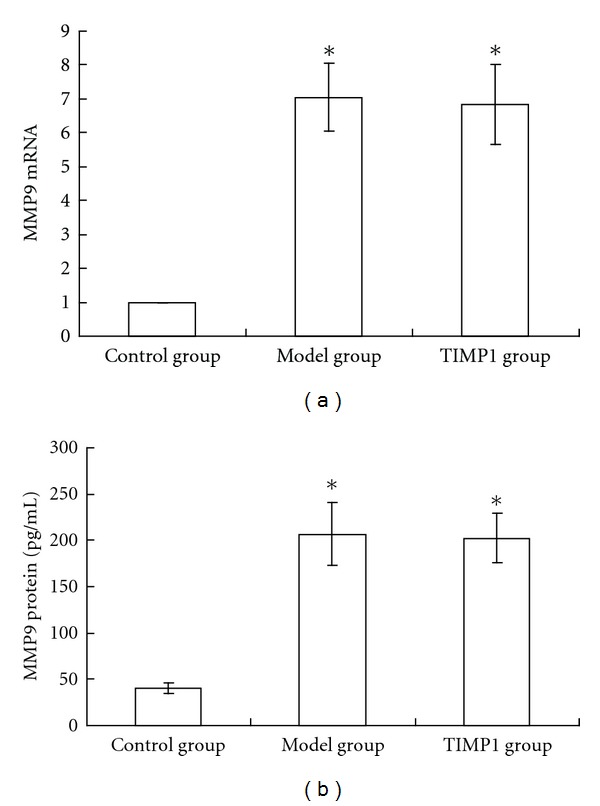
Establishment of the fibroblasts cell model of high matrix metalloproteinase 9 (MMP9) expression. The expressions of MMP9 mRNA, protein levels, and protease activity in model group were 6.05-, 4.12-, and 1.62-fold, respectively, greater than those in control group, which suggested that high expression of MMP9 was induced. When the cells were treated with tissue inhibitor of metalloproteinase 1 (TIMP1), the expression of MMP9 mRNA and protein had no statistical differences compared with model group. *P < 0.01 versus control group. Data are mean ± SEM, *n* = 5.

**Figure 2:**
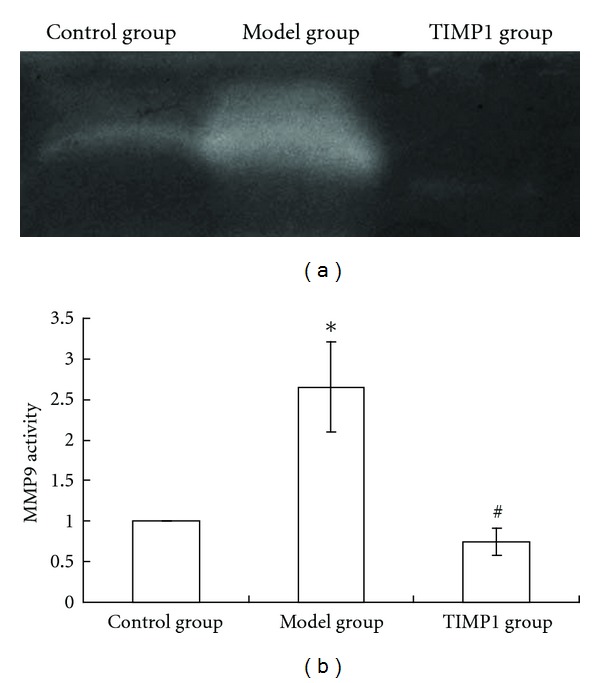
Matrix metalloproteinase 9 (MMP9) activity detected by Gelatin zymography. The activity of MMP9 in model group was 1.62-fold greater than that in control group. When the cells were treated with tissue inhibitor of metalloproteinase 1 (TIMP1), the activity of MMP9 decreased by 70.7% compared with model group. *P < 0.01 versus control group; ^#^ *P* < 0.01 versus model group. Data are mean ± SEM, *n* = 5.

High glucose and high homocysteine induced MMP9 expression obviously. mRNA and protein expression and protease activity of MMP9 in model group were 6.05-, 4.12-, and 1.62-fold, respectively, higher than those in control group (*P* < 0.01). This suggested that the fibroblasts cell model of high MMP9 expression was created successfully. When the cells were treated with TIMP1 (100 *μ*g/L), MMP9 protease activity decreased by 70.7% significantly (*P* < 0.01), while MMP9 mRNA and protein expression had no statistical differences compared with those in model group (*P* > 0.05) (Figures 1 and 2).

### 3.2. Inhibition of Fibroblasts Proliferation, Viability, and Collagen Secretion by High MMP9 Expression

Compared with those in control group, fibroblasts proliferation, viability, and collagen secretion in model group were inhibited with 29.8% of S-phase cell ratio, 18.1% of proliferation index, 23.3% of cell viability, and 68.7% of collagen secretion (*P* < 0.01, resp.). After treatment with TIMP1, the inhibition reduced compared with those in model group (*P* < 0.05) (Table 1).

### 3.3. Inhibition of Fibroblasts Horizontal Migration by High MMP9 Expression

**Figure 3:**
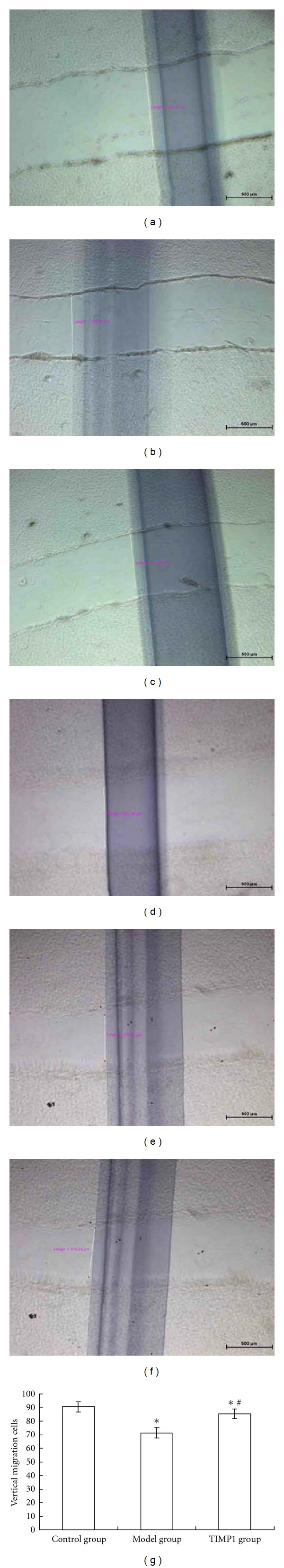
Scratch test of each group. (a) Control group 0 h (×40); (b) model group 0 h (×40); (c) tissue inhibitor of metalloproteinase 1 (TIMP1) group 0 h (×40); (d) control group 6 h (×40); (e) model group 6 h (×40); (f) TIMP1 group 6 h (×40); (g) histogram. The horizontal migration rate of fibroblasts in model group was lower than that in control group with the inhibition rate being 45.0%. After treatment with TIMP1, the horizontal migration rate increased compared with that in model group, but failed to recover to the level of control group. *P < 0.01 versus control group; ^#^ *P* < 0.01 versus model group. Data are mean ± SEM, *n* = 5.

The horizontal migration rate of fibroblasts in model group (21.3 ± 2.1)% was lower than that in control group (38.7 ± 2.6)% with the inhibition rate being 45.0% (*P* < 0.01). After treatment with TIMP1, the horizontal migration rate (31.5 ± 2.7)% increased compared with that in model group but failed to recover to the level of control group (*P* < 0.01, resp.) (Figure 3).

### 3.4. Inhibition of Fibroblasts Vertical Migration by High MMP9 Expression

**Figure 4:**
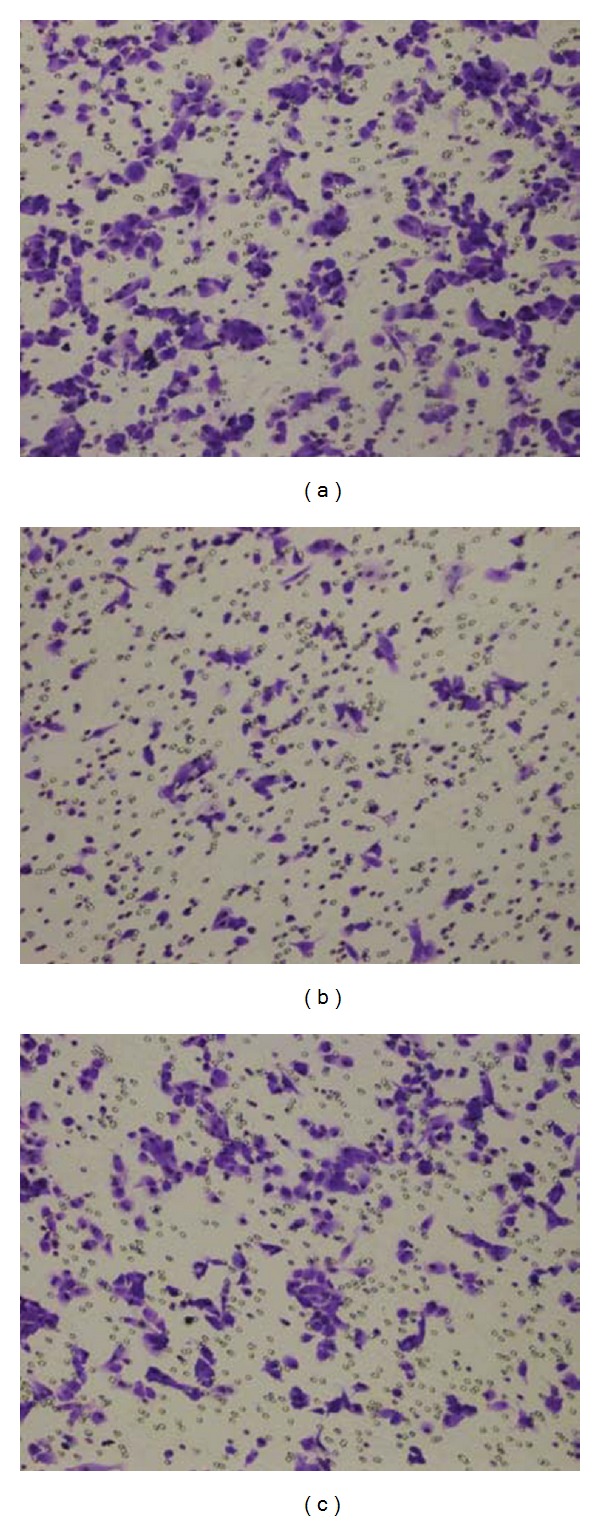
Vertical migration of fibroblasts. (a) Control group (×100); (b) model group (×100); (c) tissue inhibitor of metalloproteinase 1 (TIMP1) group (×100). Compared with control group, the vertical migration of cells decreased by 21.4% in model group. After treatment with TIMP1, the vertical migration of cells increased compared with that in model group but failed to recover to the level of control group.

Compared with control group (90.6 ± 3.8), the vertical migration of cells decreased by 21.4% in model group (71.2 ± 3.8) (*P* < 0.01). After treatment with TIMP1, the vertical migration of cells (85.3 ± 3.7) increased compared with that in model group but failed to recover to the level of control group (*P* < 0.01) (Figure 4).

## 4. Discussion

Fibroblasts are major repair cells in the skin, which account for 40% to 60% of total cells. The biological effects of fibroblasts play vital roles in wound healing [19]. Scholars have found that the DNA synthesis of skin fibroblasts significantly decreased, while apoptosis increased, in patients with diabetes, which indicates that the proliferation of fibroblasts was inhibited in the pathological statement of diabetes [20]. Studies in rats demonstrated similar results to those in humans [21]. However, whether the changes in biological behaviors of fibroblasts are related to high MMP9 expression in the diabetes is still unknown.

To avoid the complicating factors in vivo, high concentrations of glucose and homocysteine were used to mimic the local environment of diabetic skin [12]. Results of this study showed that MMP9 expressions of fibroblasts increased when cultured in high glucose and high homocysteine medium. The expressions of MMP9 mRNA, protein levels, and protease activity in high-glucose and high-homocysteine-treated group were 6.05-, 4.12-, and 1.62-fold, respectively, higher than those in control group, which suggested that high expression of MMP9 was induced.

At the same time, the S-phase cell ratio, proliferation index and cell viability in high-glucose and high-homocysteine-treated group decreased significantly compared with those in control group. After treatment by TIMP1, expression of MMP9 mRNA and protein levels had no statistical change, but activity of MMP9 protein was inhibited significantly, together with the increase of S-phase cell ratio, proliferation index, and cell viability. These results suggested that the inhibition of fibroblasts proliferation and viability were likely related with the high activity of MMP9.

Wound healing depends on the fibroblasts proliferation, migration, granulation tissue formation, collagen secretion, and collagen-based scar formation, which need the participation of many different cells [22]. As fibroblasts are the most important cells of collagen synthesis [23], barriers of proliferation and vitality inevitably have negative effects on new collagen synthesis and wound healing. The present study found that, compared with that in control group, the amount of hydroxyproline in model group decreased by 68.7%. After treatment by TIMP1, the hydroxyproline synthesis increased. The result suggested that in the diabetic status, not only did high protease activity of MMP9 increase the degradation of extracellular matrix [24] but also the collagen synthesis was decreased because of the decrease in the number and activity of fibroblasts. Thus the collagen metabolism was kept in negative balances and will ultimately delay the healing of diabetic wounds.

Migration is another biological property of fibroblasts [25]. It is generally accepted that there is a positive correlation between expression of MMP9 and migration behavior of cells [26, 27]. In our study, it is very interesting that both the horizontal and vertical migration abilities of fibroblasts were significantly decreased in model group. The possible mechanisms of this phenomenon may be as follows. Firstly, high expression of MMP9 may result in excessive matrix protein degradation, which is necessary to support the migration of fibroblasts. Our previous data showed that the expression level of MMP9 of the skin was significantly enhanced during wound healing in diabetic rats [4–6]. Reiss et al. [28] further demonstrated that exogenous MMP9 directly delayed wound healing in a mouse model. Secondly, increased activity of MMP9 is capable of cleaving nonmatrix proteins [29], which are essential to the migration of cells [30]. Kyriakides et al. [31] found that antibody-based blockade of MMP9 function or MMP9 deficiency retarded migration, and the rate of reepithelialization was significantly delayed in MMP9 knockout mice when compared with wide-type mice. In this study, the migration ability of fibroblasts in model group decreased, which will prevent fibroblasts from crawling to the site of wound in time to play their roles. Because the inhibition of fibroblasts proliferation, viability, and collagen synthesis, even if they migrate to the site of wound, they still cannot function normally—they cannot proliferate efficiently to make up adequate cells and they can not produce enough extracellular matrix and cell factors, so that the normal cycle of wound healing is broken, and this will cause the disorder of wound healing.

## 5. Conclusion

In our previous studies, fibroblasts exhibited an upregulation of MMP9 as a result of high glucose and high homocysteine incubation [10, 11]. The present study further proved that the biological behaviors of rat dermal fibroblasts can be inhibited by high level of MMP9, and the inhibited effect can be reduced by TIMP1, which can inhibit the activity of MMP9. So we believe that inhibiting the activity of MMP9 may be a feasible way to accelerate the healing of diabetes skin ulcers. However, the delayed wound healing is a complicated procedure, and the potential involvement of other MMP and TIMP family members and related cytokines still needs further investigation.

Deficiencies: The present study is performed in vitro, and the results of this research remain to be further confirmed in future animal experiments.

## Figures and Tables

**Table 1:** Proliferation, viability, and collagen secretion of fibroblasts.

Group	S-phase cell ratio (%)	Proliferation index (%)	CCK-8 OD value	Hydroxyproline (pg/mL)
Control group	9.31 ± 0.24	13.8 ± 0.5	1.76 ± 0.13	1126.4 ± 62.7
Model group	6.54 ± 0.29*	11.3 ± 0.6*	1.35 ± 0.12*	352.8 ± 60.6*
TIMP1 group	7.75 ± 0.27^∗#^	12.5 ± 0.5^∗§^	1.64 ± 0.14^#^	828.6 ± 58.9^∗#^

TIMP1: tissue inhibitor of metalloproteinase 1; *P < 0.01 versus control group; ^§^ *P* < 0.05; ^#^ *P* < 0.01 versus model group. Data are mean ± SEM, *n* = 5.
